# The Heart Is an Early Target of Anthrax Lethal Toxin in Mice: A Protective Role for Neuronal Nitric Oxide Synthase (nNOS)

**DOI:** 10.1371/journal.ppat.1000456

**Published:** 2009-05-29

**Authors:** Mahtab Moayeri, Devorah Crown, David W. Dorward, Don Gardner, Jerrold M. Ward, Yan Li, Xizhong Cui, Peter Eichacker, Stephen H. Leppla

**Affiliations:** 1 Bacterial Toxins and Therapeutics Section, National Institute of Allergy and Infectious Diseases, National Institutes of Health, Bethesda, Maryland, United States of America; 2 Rocky Mountain Laboratories, National Institute of Allergy and Infectious Diseases, National Institutes of Health, Hamilton, Montana, United States of America; 3 Infectious Diseases Pathogenesis Section, Comparative Medicine Branch, National Institute of Allergy and Infectious Diseases, National Institutes of Health, Bethesda, Maryland, United States of America; 4 Critical Care Medicine Department, National Institutes of Health Clinical Center, National Institutes of Health, Bethesda, Maryland, United States of America; The University of Texas-Houston Medical School, United States of America

## Abstract

Anthrax lethal toxin (LT) induces vascular insufficiency in experimental animals through unknown mechanisms. In this study, we show that neuronal nitric oxide synthase (nNOS) deficiency in mice causes strikingly increased sensitivity to LT, while deficiencies in the two other NOS enzymes (iNOS and eNOS) have no effect on LT-mediated mortality. The increased sensitivity of nNOS−/− mice was independent of macrophage sensitivity to toxin, or cytokine responses, and could be replicated in nNOS-sufficient wild-type (WT) mice through pharmacological inhibition of the enzyme with 7-nitroindazole. Histopathological analyses showed that LT induced architectural changes in heart morphology of nNOS−/− mice, with rapid appearance of novel inter-fiber spaces but no associated apoptosis of cardiomyocytes. LT-treated WT mice had no histopathology observed at the light microscopy level. Electron microscopic analyses of LT-treated mice, however, revealed striking pathological changes in the hearts of both nNOS−/− and WT mice, varying only in severity and timing. Endothelial/capillary necrosis and degeneration, inter-myocyte edema, myofilament and mitochondrial degeneration, and altered sarcoplasmic reticulum cisternae were observed in both LT-treated WT and nNOS−/− mice. Furthermore, multiple biomarkers of cardiac injury (myoglobin, cardiac troponin-I, and heart fatty acid binding protein) were elevated in LT-treated mice very rapidly (by 6 h after LT injection) and reached concentrations rarely reported in mice. Cardiac protective nitrite therapy and allopurinol therapy did not have beneficial effects in LT-treated mice. Surprisingly, the potent nitric oxide scavenger, carboxy-PTIO, showed some protective effect against LT. Echocardiography on LT-treated mice indicated an average reduction in ejection fraction following LT treatment in both nNOS−/− and WT mice, indicative of decreased contractile function in the heart. We report the heart as an early target of LT in mice and discuss a protective role for nNOS against LT-mediated cardiac damage.

## Author Summary

Injection of pure anthrax lethal toxin (LT) at the levels found in terminally-ill, anthrax-infected animals induces an atypical vascular collapse in several experimental animals without the associated hemorrhagic manifestations and disseminated intravascular coagulopathies seen in bacteria-induced shock states. Nevertheless, LT alone produces the pleural edema that is a classic manifestation of anthrax. All the histopathological changes previously observed in LT-treated mice can be explained as predictable sequelae of vascular insufficiency. In this report, electron microscopic analysis was used to identify rapid and striking pathological changes in endothelial cells, myocytes, mitochondria, and the sarcoplasmic reticulum cisternae in the hearts of LT-challenged mice. These morphological changes were paralleled by release of very high levels of multiple cardiac injury biomarkers. LT-induced pathological changes occurred earlier and with markedly higher severity in nNOS knockout mice compared to wild-type or iNOS or eNOS knockout mice, reflecting the well-established role of nNOS in maintenance of cardiac function, and suggesting that nitric oxide produced by nNOS counteracts the damage induced by LT.

## Introduction

Anthrax toxin, the major virulence factor of *Bacillus anthracis*, consists of three polypeptides: Edema Factor (EF), Lethal Factor (LF) and Protective Antigen (PA). LF is toxic in animal models when combined with the receptor-binding PA, which facilitates LF entry into cells [1]. Mouse strains have been shown to have a range of susceptibilities to anthrax lethal toxin (LT, the combination of PA and LF) [2] and succumb displaying atypical shock-like symptoms without classic systemic hemorrhagic manifestations [3]. LT induces rapid lysis of macrophages isolated from some inbred mouse strains, and in vivo macrophage sensitivity to LT can contribute to a cytokine storm and more rapid death in certain strains. LT susceptibility, however, is distinctly independent of macrophage sensitivity in other mouse strains [2]. LT-induced events lead to death of mice in 2–3 days, while some rat strains die in as little as 37 min when subjected to the same dose of toxin [4],[5]. This extremely rapid death in rats, which is dependent on PA and transport of LF to the cellular cytoplasm, is accompanied by a rapid accumulation of pleural fluid. Although LF is known to cleave most of the mitogen-activated protein kinase kinases (MAPKKs) [6],[7], the molecular basis for LT-mediated effects leading to shock in animals is currently unknown.

Nitric oxide (NO) is one of the most potent modulators of vascular function in vivo and its production by NO synthases is often highly increased during infections. The role of NO in LT- or *Bacillus anthracis*-mediated pathogenesis has been investigated in a number of studies. We previously tested inducible NO synthase (iNOS) knockout mice (iNOS−/−) for susceptibility to LT and found no difference in LT sensitivity relative to WT controls [2]. Surprisingly, iNOS−/− mice also display similar susceptibility to wild type controls in *B. anthracis* infection in mice [8]. In the rat LT toxicity model, in striking contrast to endotoxic shock, LT induces circulatory shock with no associated changes in NO concentrations [9]. While it has been reported that *B. anthracis* infection can induce NO production in macrophages [10], LT actually has inhibitory effects on NO induction by iNOS through many classic stimuli such as lipopolysaccharide (LPS) [11]. LT itself does not induce NO production in macrophage cell lines or peritoneal macrophages and changes in NO circulatory levels were not detected in LT-treated mice ([2] and data not shown).

The rapid and impressive changes that occur in NO concentrations during bacterial infections are generally attributed to the action of the inducible NO synthase isoform (iNOS). After previous studies did not identify a role for iNOS in LT-mediated effects [2], we chose to extend these studies to examine the role of the constitutive NO synthases in LT-mediated pathogenesis. Knockout mice lacking either endothelial NO synthase (eNOS) or neuronal NO synthase (nNOS) have been useful for delineating the roles of these important enzymes in NO-mediated events. In the current study we report on the increased sensitivity of nNOS−/− mice to toxin when compared to iNOS−/− and eNOS−/− mice, as well as to WT mice, suggesting a protective role for nNOS in LT-mediated pathogenesis. Histopathological, electron microscopy and heart biomarker analyses revealed that LT results in rapid and striking pathology in the murine heart and that nNOS may provide a degree of protection against LT-mediated cardiac effects.

## Results

### nNOS−/− mice have significantly increased sensitivity to LT

**Figure 1:**
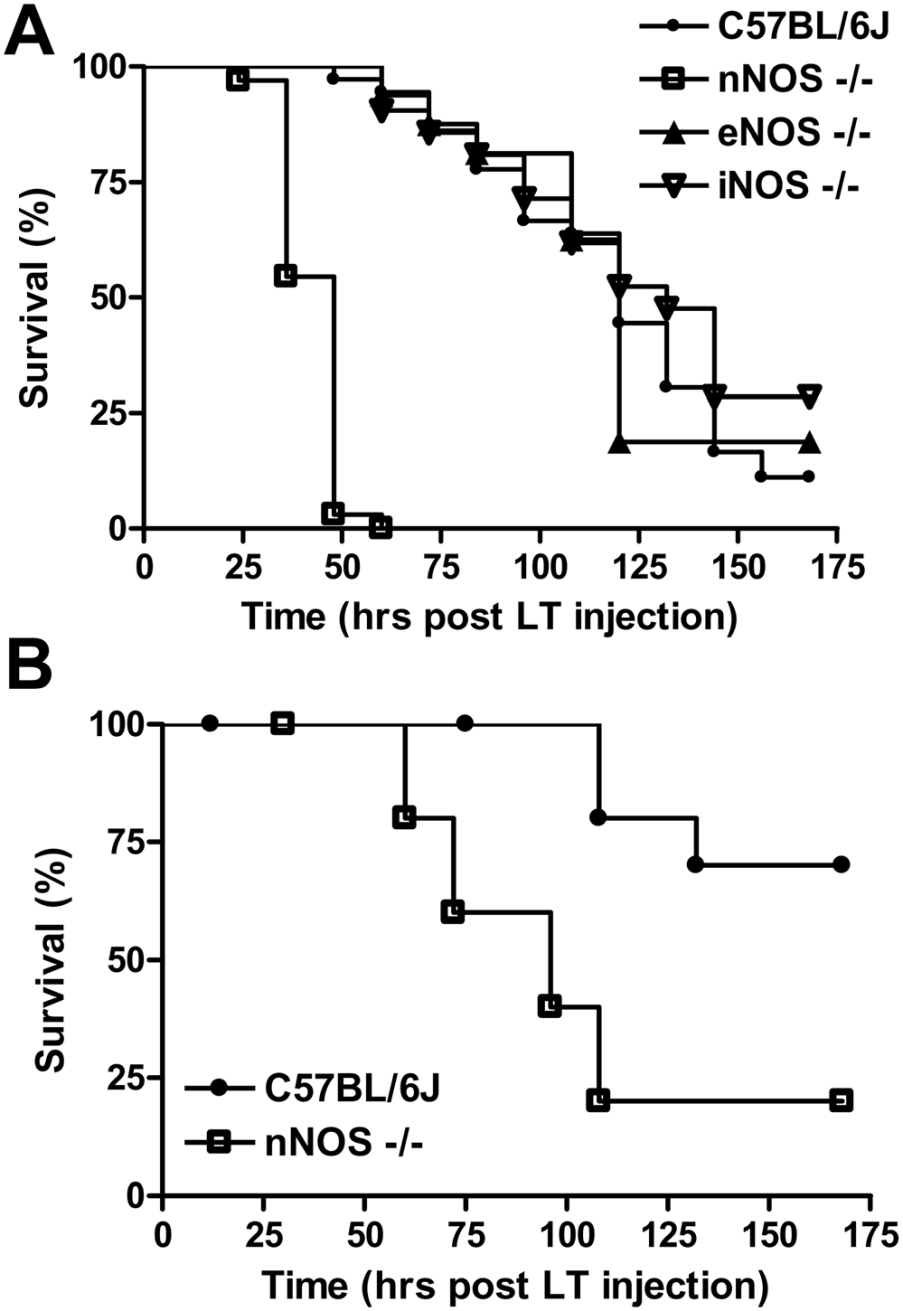
nNOS−/− mice are more susceptible to LT. nNOS−/−, eNOS−/−, iNOS−/−, and C57BL6/J mice were treated with LT ((A) 100 µg IP, or (B) 40 µg IP) and monitored for survival. The experiment shown in (A) represents data from four independent toxin challenge experiments that included all four mouse strains. Eleven additional independent toxin challenge experiments that included controls and nNOS−/− mice also yielded the same results. The n values for panel (A) are as follows: C57BL/6J (n =  36), nNOS−/− (n = 33), eNOS−/− (n =  16), iNOS−/− (n = 21). Logrank test P-values for comparison of nNOS−/− mouse survival curves with each of the eNOS−/−, iNOS−/−, and C57BL/6J mouse survival curves are <0.0001 for panel A. The experiment in (B) (n = 10 for each strain) is a single experiment representative of two independent experiments.

Because LT targets the vasculature [3],[9],[12],[13] and NO plays a key role in control of vascular function, we previously tested iNOS−/− mice, but found no change in LT sensitivity [2]. To determine whether the other NO synthases control LT susceptibility, we compared iNOS, eNOS and nNOS−/− mice and their corresponding parental strains to challenge with a single dose of LT (100 µg). The iNOS−/− and eNOS−/− mice did not show any significant difference in susceptibility to LT compared to their C57BL/6J (Figure 1A) or B6.129SF2/J (data not shown) parental controls, with over 50% of the animals succumbing by 120 h (Figure 1A and data not shown). However, the nNOS−/− mice were uniquely sensitive to LT with 50% of mice succumbing by 30 h. More than 95% of these animals were dead by 48 h after a single challenge of LT (100 µg). At the lower challenge dose of 40 µg, 80% of the nNOS−/− mice died between 50 and 108 h (Figure 1B), while the 30% of control animals that succumbed only started doing so at 108 h. In general, malaise symptoms were apparent in toxin-challenged nNOS mice as early as 12 h after injection while control animals rarely showed signs of malaise until 60 h. Thus, nNOS-deficient mice were clearly more sensitive to LT than their wild type counterparts. Treatment with PA alone or PA and enzymatically inactive LF is not lethal to mice (data not shown).

### Increased sensitivity of nNOS−/− mice is not due to altered macrophage sensitivity or cytokine responses

**Figure 2:**
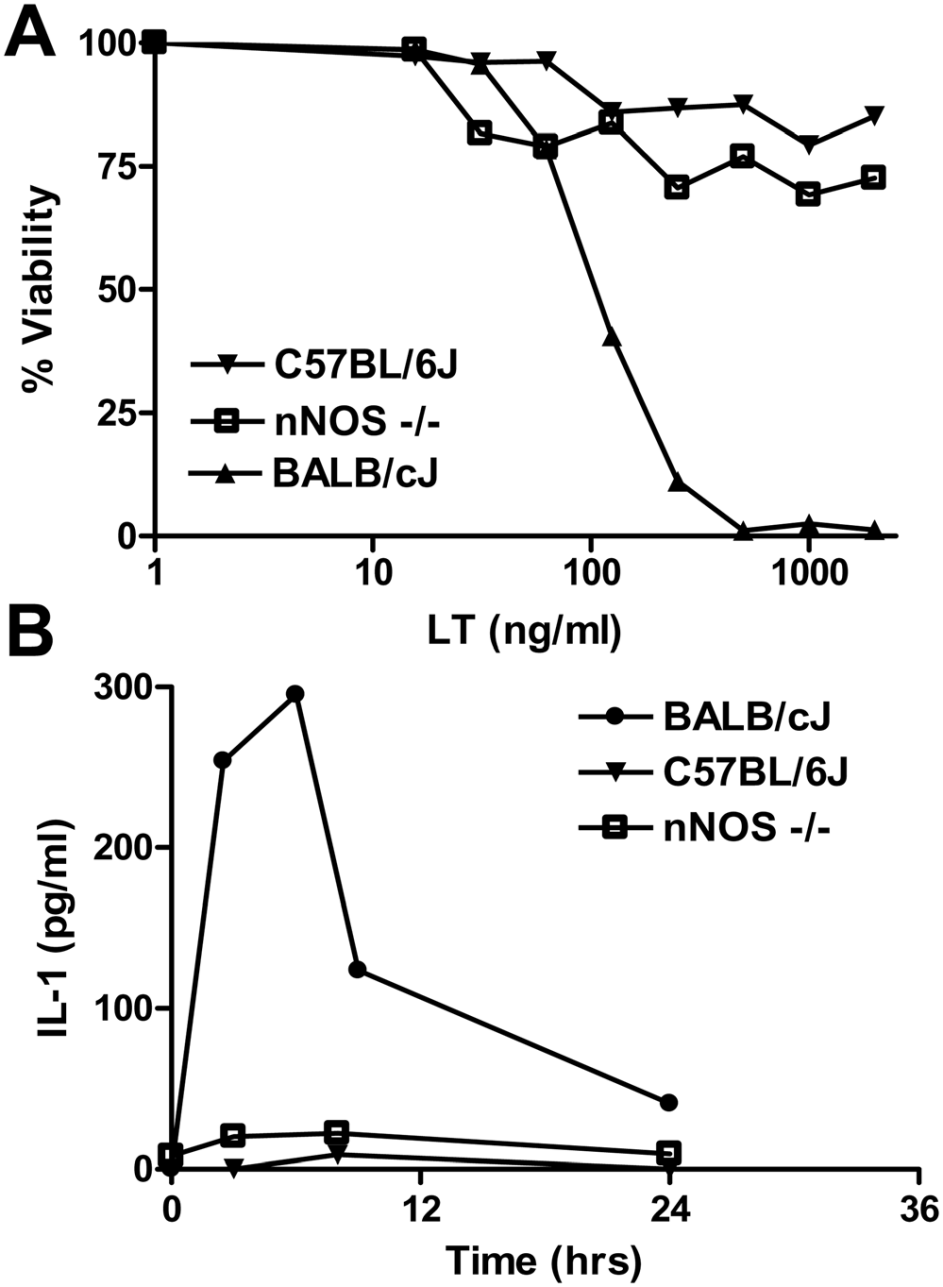
nNOS−/− mouse macrophages are not sensitive to LT and nNOS−/− mice do not release IL-1β following LT treatment. (A) BMDMs derived from C57BL/6J, nNOS−/− and Balb/cJ mice were treated with varying doses of LT for 2 h and viability assessed by MTT staining. Percent viability is expressed relative to untreated controls. (B) Balb/cJ, C57BL/6J, and nNOS−/− mice were treated with LT (100 µg IP) and bled at four time points after LT injection (n = 2 mice/time point). IL-1β level in serum was assessed by ELISA.

We previously showed that while macrophage sensitivity to LT in mice is not a direct correlate to animal sensitivity for a number of strains, the presence of sensitive macrophages in the normally more resistant C57BL/6J strain (which harbors LT-resistant macrophages) made the mice as susceptible as Balb/cJ mice (which harbor LT-sensitive macrophages) [2]. Thus in some strains, macrophage sensitivity contributes to a more rapid onset of malaise and death. Macrophage sensitivity in mice was also shown to correlate with a rapid release of IL-1β from lysed circulating macrophages, and a subsequent rapid induction of a number of different cytokines over a 6 h period, followed by drop to baseline levels by 24–30 h [2],[3]. We tested whether nNOS could potentially play a role in macrophage sensitivity to LT resulting in the sensitization of mice. Figure 2A shows that macrophages from nNOS−/− mice are resistant to LT in a manner similar to the parental C57BL/6J strain. Accordingly, no cytokine burst (IL-1β, MIP-1, IL-6) was observed in these animals following LT treatment (Figure 2B and data not shown), indicating that nNOS deficiency was not leading to increased LT sensitivity through altered inflammatory cytokine responses as has been suggested for other shock models [14].

### 7-nitroindazole sensitizes wild-type mice to LT

**Figure 3:**
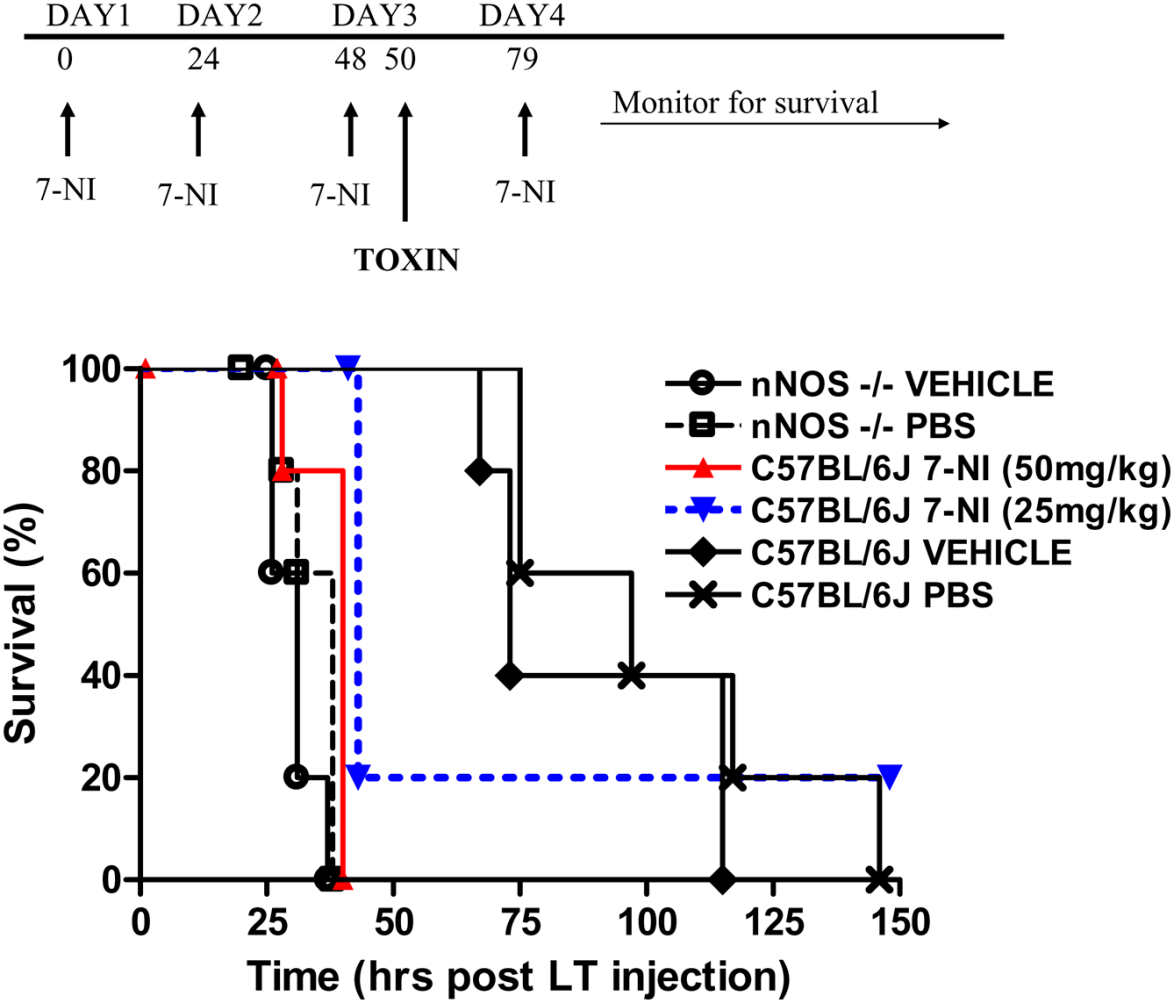
7-NI sensitizes nNOS-sufficient WT mice to LT. C57BL/6J or nNOS−/− mice were treated with 7-NI (50 mg/kg or 25 mg/kg), PBS, or drug vehicle (10% DMSO) according to the schedule shown in the top panel. LT was administered on day 3 and animals were followed for survival (n = 5 for each group). Data is representative of two similar experiments. Logrank test P-values were <0.005 when comparing survival curves for C57BL/6J /25 mg/kg+LT, C57BL/6J /50 mg/kg+LT, nNOS−/−/PBS+LT, and nNOS−/−/vehicle+LT mouse groups to either the C57BL/6J/PBS or C57BL/6J/vehicle mouse groups.

We next asked if the selective nNOS inhibitor 7-nitroindazole (7-NI) could replicate the sensitivity phenotype seen in nNOS−/− mice. Groups of C57BL/6J mice were treated with 25 mg/kg or 50 mg/kg of 7-NI once a day for three days prior to challenge with a single dose of LT (100 µg IP) on the third day. Control C57BL/6J and nNOS−/− groups were treated with the vehicle (10% DMSO) used for drug delivery, or with PBS on the same schedule and challenged with LT. A final (fourth) dose of drug or vehicle was also given on the day following toxin challenge. Figure 3 shows the experimental schedule (top panel) and the survival results, which clearly show that 7-NI sensitizes the WT mice to LT. Sensitivity levels almost identical to nNOS−/− mice were seen following 7-NI treatment of WT mice.

### Histopathology analysis of LT-treated nNOS−/− mice

**Figure 4:**
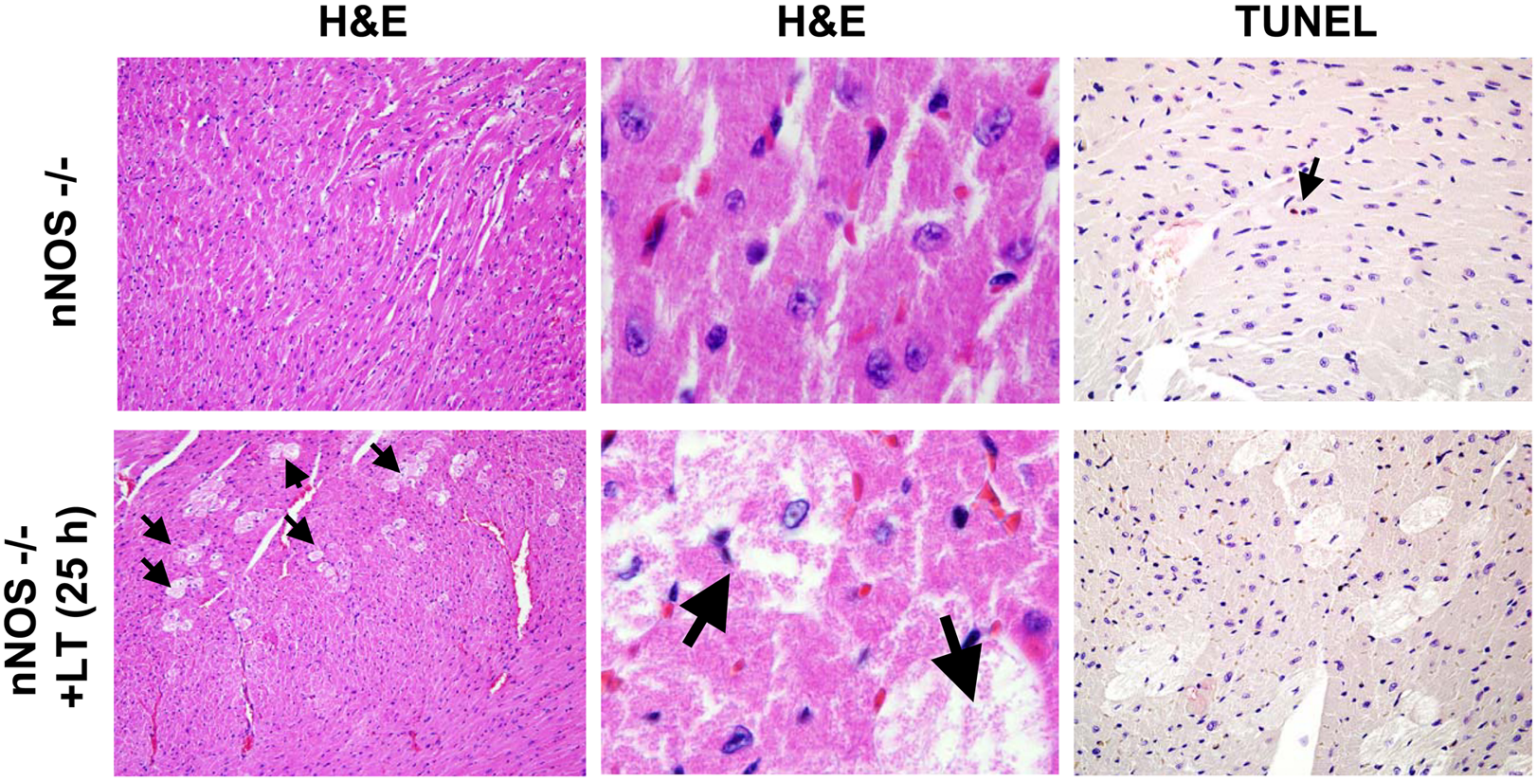
Histopathological analysis of LT-treated hearts in nNOS−/− mice. Left and central panels shown H&E analysis (10× and 100×) of heart sections from mice treated with PBS (top) or 25 h after LT (100 µg IP) treatment (bottom). Arrows point to the swollen pale cardiomyocytes seen only in toxin-treated mice. Right panels are TUNEL stain (20×) of mouse cardiac tissue from the same untreated (top) and LT-treated (bottom) mice. Single arrow points to a single TUNEL-positive cell in the untreated control.

LT treatment of mice generally produces few histopathological changes besides those considered to be sequelae of the vascular insufficiency [3]. We previously described changes in tissues such as the liver or bone marrow in mice as being due to compromised oxygenation, since the severity increased with distance from blood vessels [3]. To see if any novel pathological events occur in nNOS−/− mice treated with LT, we performed histopathological analyses on H&E-stained lung, heart, aorta, brain, kidney, spleen, liver and bone marrow obtained from knockout and control mice treated with LT for 7 h, 15 h, and a range of times between 23–36 h when different grades of malaise severity were noted. At all these times the C57BL/6J mice used as matching time point controls were not ill, while nNOS−/− mouse malaise grades ranged from (+) to (+++ = moribund). No histopathological changes were found in LT-treated mice compared to PA-treated, PBS-treated or untreated controls for the WT strain in any tissues. The only LT-induced pathological changes found in the nNOS−/− mice were in the hearts of mice having marked malaise symptoms at later time points. The hearts from these LT-treated nNOS−/− mice showed novel inter-fiber vacuole-like spaces in the cardiac muscle tissue, with grainy punctuate staining in the tissue, possibly indicative of altered (swollen) mitochondria. The spaces also appeared to hold granular content which were suggestive of released mitochondria or cell debris (Figure 4). LT-treated WT mice did not indicate any histopathological changes at any time point and resembled the untreated nNOS−/− mice shown in Figure 4 throughout the course of the experiment (data not shown).

Because apoptosis has been shown to be modulated by NO in the heart [15],[16], we asked whether the heart tissue changes observed in toxin-treated nNOS−/− mice could be accompanied by apoptotic cell death. TUNEL staining, however, indicated that with the exception of the occasional lone TUNEL-positive cell (in this case in the untreated mouse heart), apoptosis was not observed in the LT-treated hearts (Figure 4). Because nNOS has been shown to be up-regulated as a protective response in hypoxia and shock [17],[18], we immunostained for nNOS, to see if LT treatment actually resulted in any change in the levels of this enzyme. Strong staining was observed in the hearts of both PA-treated, untreated and LT-treated mice, with no differences evident (data not shown). Myeloperoxidase staining was used to assess neutrophil infiltration (often associated with cardiac damage), and cytochrome c oxidase I (COX1) staining was used to investigate potential loss of mitochondria from cardiomyocytes. No differences between the LT-treated and untreated samples were seen for either of these stains. Of course the COX1 was only indicative of the presence of the mitochondria and not their level of respiratory function in the hearts. Masson trichrome staining (for cardiac fibrosis and necrosis) also did not yield significant differences associated with LT treatment in a blind-test analysis (data not shown). Altogether, with the exception of the novel inter-fiber vacuole-like spaces seen in LT-treated nNOS−/− hearts, light microscopy did not show any immune cell infiltration or pathological changes.

### Cardiac injury biomarkers myoglobin, cTnI, and H-FABP are elevated with LT treatment

**Figure 5:**
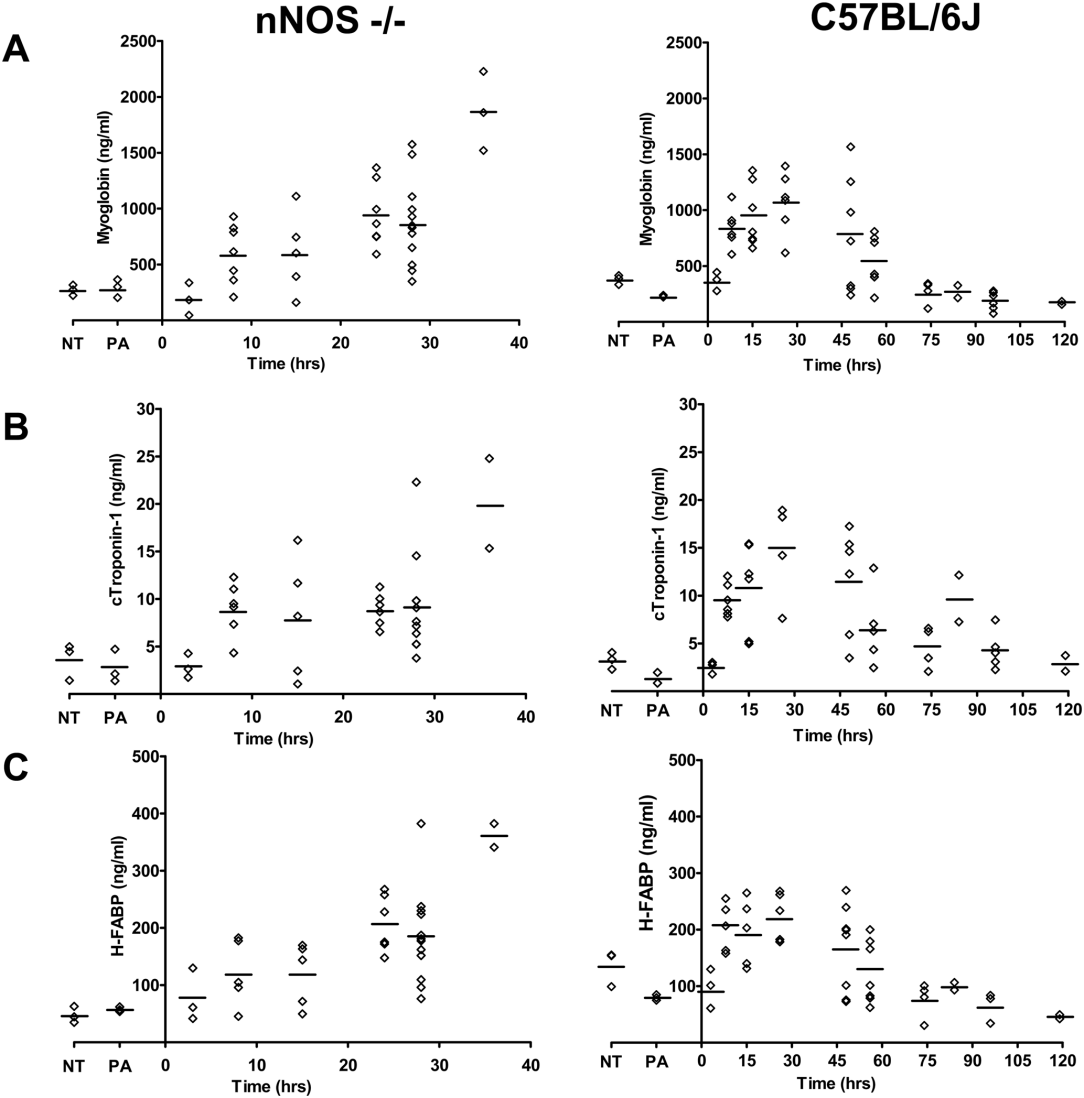
Cardiac injury biomarkers myoglobin, cTroponin-I (cTnI), and heart-type fatty acid binding protein (H-FABP) are elevated after LT treatment of mice. Groups of C57BL/6J (WT) (n = 49) and nNOS−/− mice (n = 36) were injected with a single bolus of LT (100 µg IP) and groups of mice were bled to obtain serum at various time points after injection. Three control animals were treated with PA (100 µg IP) or left untreated (NT). Levels of myoglobin (A), cTroponin-I (B) and H-FABP (C) were measured for all samples by ELISA. Each symbol represents an individual mouse; mean measurements for each group are shown.

Because nNOS is extremely important in cardiac function (for reviews see [19]–[21] we measured the levels of cardiac damage biomarkers in circulation following toxin treatment. Myoglobin is one of the first and most sensitive markers for detection of low levels of myocardial necrosis [22] but because it also exists in skeletal muscle, it must be used in combination with other heart-specific markers. Cardiac troponin-I (c-TnI), a regulator of contractile function, is the universally preferred biomarker for monitoring cardiotoxicity [23],[24] and a wide range of cardiac injuries [25]. High levels have been shown to appear in experimental animals from 2–6 h after cardiac injury [24],[26]. Heart type fatty acid binding protein (H-FABP) is a highly cardiac muscle-specific cytoplasmic peptide that can be detected even earlier than c-TnI [27],[28]. We measured myoglobin, c-Tn and H-FABP protein levels in serum from nNOS−/− and C57BL/6J mice at various times post-LT challenge. A rapid dramatic increase in all three proteins was found in the circulation of LT-treated nNOS−/− mice beginning at very early time points, 6–10 h after toxin treatment (Figure 5A–5C, left panels). Amazingly, a similar striking early increase in these proteins was also seen in the WT mice following LT challenge (Figure 5A–5C, right panels). The cardiac damage demonstrated by these measurements is one of the earliest events we or others have observed in LT-challenged WT mice. Protein biomarker levels continued to increase or remain high in the nNOS−/− mice until the mice met moribundity criteria requiring euthanasia (25–36 h) (Figure 5A–5C, left panels). In the C57BL/6J mice, however, the increased biomarker concentrations seen in the first 36 h were reversed after 36 h, with an actual reduction to control or below control levels of these proteins at late time points when the mice showed more severe malaise symptoms (Figure 5A–5C, right panels). Finding concentrations of c-TnI exceeding 10 ng/ml in response to LT treatment was particularly striking, as the maximum published levels of c-TnI in mice following induced myocardial injury or toxicity in various studies are 3.5–9 ng/ml [24],[26].

### Electron microscopy of hearts from LT-treated mice

**Figure 6:**
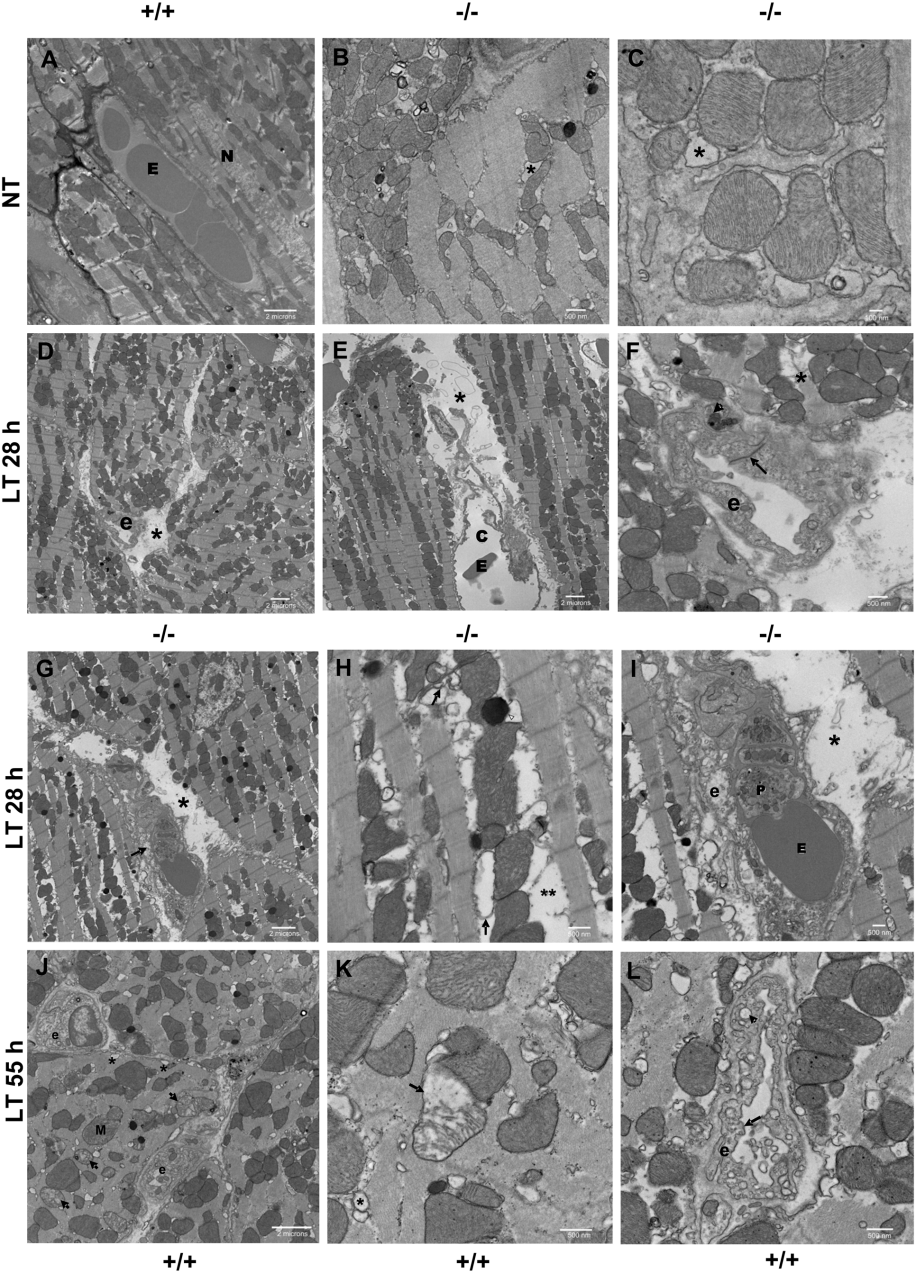
Electron microscopy of LT-treated nNOS−/− and WT mouse hearts. Groups of C57BL/6J (WT) (n = 10) and nNOS−/− mice (n = 10) were injected with a single bolus of LT (100 µg IP) and hearts harvested at 12 h (when both strains show no malaise symptoms) or after moderate to marked malaise symptoms (28–30 h for nNOS−/− and 55–60 h for C57BL/6J mice). Three control mice were treated with PA (100 µg IP) or left untreated (NT). Images representative of each group are shown at differing magnifications as shown by the scale markers. (A) Untreated C57BL/6J with intact myocytes and endothelium (E = erythrocyte, N = myocyte nucleus). (B–C) Untreated nNOS−/− mice with swollen sarcoplasmic reticulum (SR) cisternae (*) and general mitochondrial swelling. (D–I) nNOS−/− mice, +LT, 28 h: (D) Capillary necrosis (e = endothelial cell) and inter-fiber edema (*). (E) necrotic capillary with expansion of lumen (c), inter-fiber edema (*), and debris (E =  erythrocyte). (F) endothelial necrosis (pyknotic endothelial nucleus, arrowhead); increased density of endothelial intercellular junction (arrow); fragmented myofilament (*)(e = endothelial cell). (G) Capillary necrosis (arrow); inter-fiber edema and cell debris (*). (H) shows detail of (G): myofilament degeneration with separation of myofibrils from mitochondria (**), swollen SR cisternae and transverse (T) tubules (arrow); arrowhead points to lipid. (I) Further detail of (G): endothelial and capillary necrosis with intraluminal erythrocyte (E) and platelet (P); inter-fiber edema and cell debris (*); (e = endothelial cell). (J–L) C57BL/6J mice+LT, 55 h: (J) Endothelial cell swelling, mitochondrial degeneration (arrows), multiple swollen SR cisternae (*); M = mitochondria and e = endothelial cell. (K) Detail of degenerate mitochondrion (arrowhead) and swollen SR (*). (L) Endothelial degeneration; endothelial cytoplasmic swelling with increased vacuolation; irregular endothelial plasma membrane (e = endothelial cell, arrow marks plasma membrane, arrowhead points to representative abnormally enlarged cytoplasmic vacuole).

Based on the clear indications of myocardial injury and because light microscopy is not sensitive enough for observing changes in mitochondrial morphology or cardiac endothelium, we performed electron microscopy on heart sections prepared from untreated, PA-treated (i.e., a second control) or LT-treated nNOS−/− and WT mice. PA-treated mice were not different from untreated animals at any time point (data not shown). Hearts of untreated nNOS−/− mice showed some baseline differences from the WT animals (Figure 6A), including occasional swelling of sarcoplasmic reticulum (SR) cisternae and mitochondria (Figure 6B and 6C). Notably, nNOS−/− mice at later times (>24 h) after toxin treatment had distinct and striking pathological changes including endothelial necrosis, inter-fiber edema with cell debris, altered endothelial junctions and even fragmented myofilaments (Figure 6D–6I). Necrotic capillaries were commonly noted in these mice after LT treatment. Myofilament degeneration with separation of myofibrils from mitochondria was also observed (Figure 6G and 6H), clearly indicating cardiomyocyte injury in LT-treated nNOS−/− hearts. Surprisingly, the WT mice which did not exhibit early pathologies did manifest many similar pathological changes at later times after LT treatment. By 55 h post-LT treatment, the WT mice showed endothelial cell swelling and degeneration, altered endothelial junctions, mitochondrial degeneration/swelling, swollen SR cisternae and myofilament fragmentation (Figure 6J–6L). Thus, LT clearly affected both nNOS−/− and WT hearts in a similar fashion, but with delayed timing and severity in nNOS-sufficient WT mice. These findings demonstrate the ability of electron microscopy to detect important changes that could not be observed in our prior light microscopy studies and clearly identify the heart as an early LT target in vivo.

### Echocardiography of LT-treated hearts

To test cardiac function in LT-treated animals, we performed echocardiography on nNOS−/− and WT mice treated with LT for 24 h compared to untreated controls (Figure S1). Surprisingly, we found a wide range of variation in the measured parameters for each test group. As a result, statistically significant differences were found only between nNOS−/− and WT animal baseline parameters, whereas no differences were found between untreated and LT-treated mice. The left ventricle (LV) end systolic volume (Vs) and end systolic diameter measurements (Ds) differed significantly between the nNOS−/− and WT mice (P = 0.0202 and P = 0.0176, respectively). The ejection fraction (EF) and fractional shortening (FS) parameters were also different (P = 0.0325 and 0.0368, respectively), indicative of lower contractile function in nNOS−/− mice. The other parameters measured are identified in the Figure S1 legend. Previous analyses of nNOS−/− mice for cardiac function reported no differences in chamber sizes and basal contractility from those in parental C57BL/6 mice in the first two months of life (similar age to the mice used in our studies) with normal baseline ventricular performance [29]. Later conflicting reports described both increases and decreases in nNOS−/− mouse myocyte contractile function relative to WT mice [29]–[32]. While the wide variation in echocardiography measurements for each group in our studies prevented attainment of statistical significance, a general decreasing trend (based on mean values) in the EF from the LV following LT treatment in both the knockout and WT mice suggests loss of contractile function in response to LT. The lack of a more distinct change in LT-treated animals may simply be related to the observation that in other animal models, such as the canine model, EF reductions are only observed at very late time points [33]. Thus it is possible the manifestations of injury observed by electron microscopy have to progress further for dysfunction measurable by echocardiography to occur.

### Allopurinol, nitrite, and carboxy-PTIO effects on LT survival

**Figure 7:**
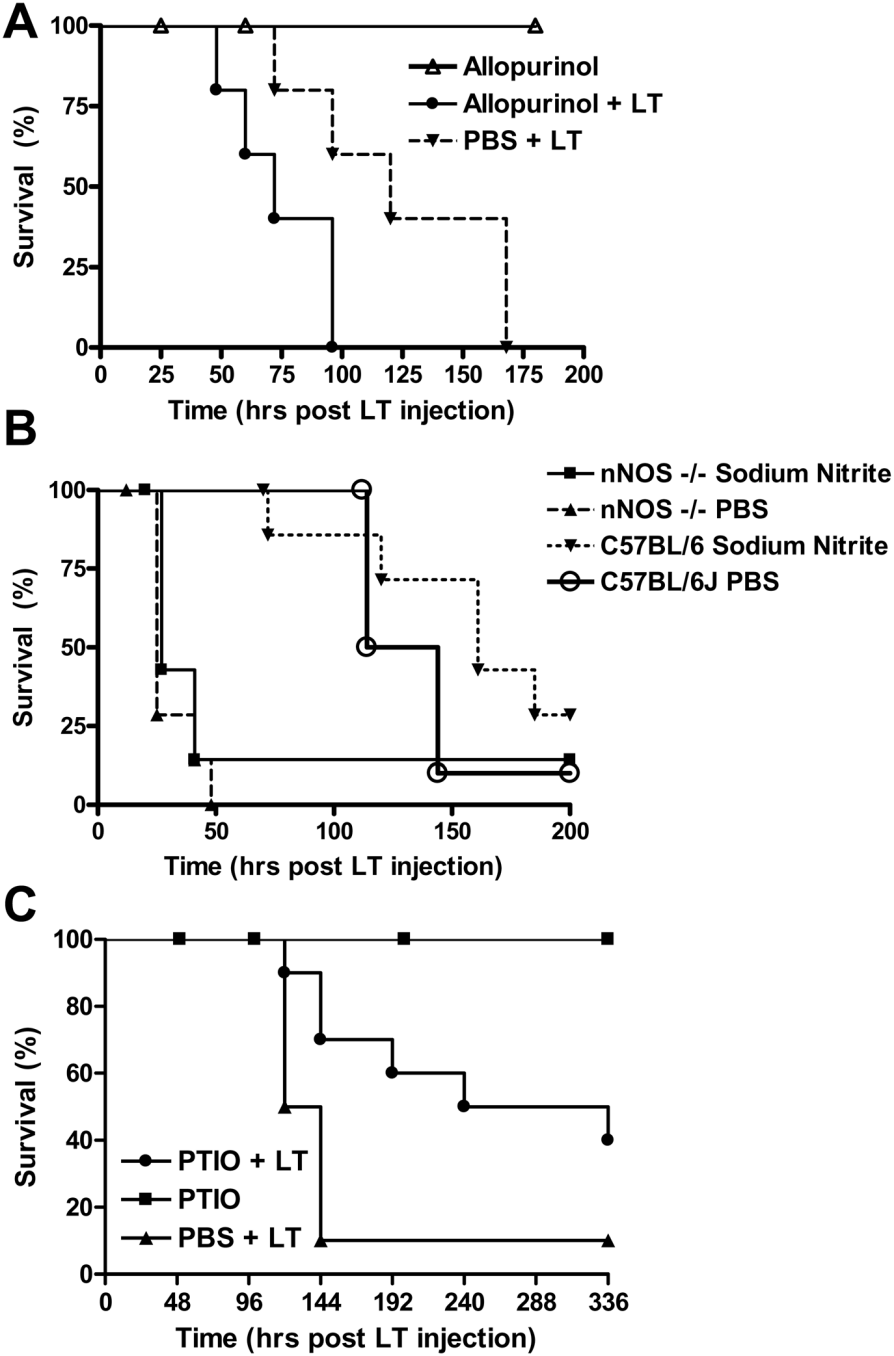
Effects of allopurinol, nitrite therapy, and carboxy-PTIO on LT-induced mortality. (A) Allopurinol treatment: C57BL/6J mice were gavaged with allopurinol (300 mg/kg, daily, for 5 days) or PBS. One drug-treated group and the PBS-treated controls were injected with LT (100 µg IP) 2 h after drug/PBS administration on day 2 while a second drug-only control group did not receive toxin. Animals were monitored for survival (n = 5/group). P-value for survival curve of allpurinol/LT vs. PBS/LT is 0.0431. (B) Nitrite Therapy: C57BL/6J and nNOS−/− mice were injected with sodium nitrite (1 mg/kg), IP, daily, 7 days) or PBS. A single bolus of LT was injected into mice 2 h after sodium nitrite administration on day 2 and animals were monitored for survival (n = 7 for all groups, except n = 10 for the C57BL/6J+LT group). Logrank-test based P-value for nNOS−/− PBS/LT vs. nNOS−/− Nitrite/LT is 0.2928 and P-value for C57BL/6J/PBS/LT vs. C57BL/6J/Nitrite/LT is 0.3907. (C) Carboxy-PTIO treatment. C57BL/6J mice were injected daily with carboxy-PTIO (100 µg/200 µl/mouse IP) and LT (100 µg) was injected 2 h after drug injection on the second day (n = 10 for both LT-treated groups and n = 3 for drug only control group). Logrank-test based P-value comparing the C57BL/6J PTIO/LT survival curve to the LT alone survival curve is 0.0248.

Since the hearts in LT-treated mice showed severe pathological changes at early time points, we decided to test therapeutic approaches used in NO-protective models. We first wanted to test the role of xanthine oxidoreductase (XOR) in the protection provided by nNOS against LT. XOR is an enzyme normally associated with reactive oxygen species (ROS) production in vascular tissues and the heart [34],[35] and its activity is thought to be directly regulated by nNOS due to their co-localization in the heart SR [36]–[38]. ROS produced by XOR can depress myocardial contractility by inhibiting contractile myofilament responsiveness to Ca^+2^. One of the ways nNOS-derived NO is thought to be cardioprotective is simply by acting as an anti-oxidant, scavenging ROS species produced by XOR in the heart during disease states [38]. Increased XOR activity in the absence of NO attenuation is associated with the pathophysiology of cardiac dysfunction [34],[39],[40] and inhibition of XOR using allopurinal has previously been shown beneficial for maintaining normal cardiac and vascular hemodynamics [39]. However since XOR itself can also act as a source of NO in the heart during hypoxia, when NOS enzymes are inhibited [35],[40], it is also possible that inhibition of XOR actually results in inhibition of protective NO production. We tested allopurinol therapy using a regimen shown effective in inhibiting XOR. Figure 7A shows that allopurinol treatment does not result in increased survival from LT, but instead results in a statistically significant exacerbation of LT effects in C57BL/6J mice, indicating that XOR activity, like nNOS, serves to protect against LT-mediated effects.

Nitrite therapy has received much attention in recent years as a cytoprotective therapy in ischemia/reperfusion injury or disease states associated with NO deficiency [41],[42]. Nitrite can be reduced to NO through numerous mechanisms at many tissue and cellular sites. We asked whether nitrite therapy could reverse the detrimental effects of nNOS deficiency in our LT pathogenesis model. We treated C57BL/6J and nNOS−/− mice with sodium nitrite (1 mg/kg mouse, IP) daily for seven days. A single bolus of LT was injected into the animals on day 2. We did not observe any statistically significant beneficial effect in mice receiving nitrite therapy compared to those treated with PBS (Figure 7B). Although this dose range and IP method of supplying nitrite has proven successful in certain ischemia/reperfusion injury models [43], it is possible that concentrations of NO adequate to protect against LT were not achieved. Perhaps a direct infusion of nitrite to the heart would be necessary to show an effect in this situation. On the other hand, it is possible that lower doses of sodium nitrite than those used here would be most effective, as has been seen in other cases [44]. However, experiments using oral delivery of nitrite at a range of doses, or IP administration of a dose of 0.1 mg/kg/mouse also did not yield significant benefit (data not shown). Thus it is likely that nNOS protection against LT is actually mediated through local, transient and rapid NO gradients that cannot be compensated through nitrite therapy. Alternatively, nNOS protective function may also require the reductase function of the enzyme, and not just the commonly studied oxygenase activity associated with NO production.

We next wanted to test if systemic scavenging of NO would result in exacerbation of LT-mediated pathology in WT mice in a manner similar to the nNOS−/− mice. Carboxy-PTIO (2-phenyl-4,4,5,5,-tetramethylimidazoline-1-oxyl-3-oxide) is a potent NO scavenger which has no direct effect on NO synthase enzymatic function. We treated WT animals with this drug prior to LT challenge. Surprisingly, instead of exacerbating LT action, carboxy-PTIO treatment of WT animals actually had a significant beneficial effect, prolonging survival of LT-treated mice. Carboxy-PTIO may provide a protective function in a manner independent of its NO scavenging ability, or target NO production by other sources. Thus, NO or other PTIO-scavenged oxidative species produced at various tissue sites (in a manner independent of nNOS) may actually have a detrimental effect in LT pathogenesis. Alternatively, PTIO scavenging of NO may increase activity of other protective enzymes. The manner in which nNOS provides a protective role against LT remains to be elucidated.

## Discussion

Anthrax LT challenge of mice induces an atypical shock without classic systemic hemorrhagic manifestations [3]. Histological studies have revealed only certain pathological changes that are considered sequelae of vascular insufficiency without providing clues to the mechanism of shock induction. Vascular dysfunction makes endothelial cells attractive candidate targets, which is also consistent with the fact that the two anthrax receptors, TEM8 and CMG2, are considered to be highly expressed on endothelial cells [45],[46]. Recently our lab has shown that LT targets tumor angiogenesis [47], further supporting direct LT-mediated effects on vasculature. Early studies on LT effects on endothelial cells in vitro reported toxin-mediated human endothelial cell apoptosis over the course of days [48],[49]. Our laboratory and others have been unable to induce LT-mediated endothelial cell death in vitro (data not shown and [50]) but endothelial barrier dysfunction is induced by the toxin in a manner independent of cell death [50]. The data presented here provides the first direct evidence of profound LT-mediated effects on the heart and specifically on cardiac vasculature, presenting this organ as a key early target for LT function.

The heart was also recently suggested as a potential target of LT in rats [12],[51]. While LT is lethal in mice over a course of 2–4 days, some rat strains die in as little as 37 min [4],[5]. Studies on LT-induced shock in toxin-infused rats (which succumb at a slower rate over 24 h) have also shown shock accompanied by hypotension, lactic acidosis and an accumulation of pleural fluid, with no associated histopathological changes, no benefit from fluid therapy, and therefore, in summary, distinct differences from classic cytokine-associated endotoxic shock [9],[52],[53]. Assessment of vascular function parameters in LT-treated rats pointed to altered cardiomyocyte function associated with acute heart failure, suggesting that the rapid and striking pulmonary edema occurs as a secondary manifestation of the failing heart [12]. Specifically, rapid myocardial dysfunction in rats was shown as a reduction of left ventricular systolic function following a single LT bolus injection [51].

The dramatic pathological changes seen in the mouse heart following LT treatment in the current study are clearly independent of macrophage sensitivity and cytokine responses since C57BL/6J mice harbor LT-resistant macrophage and have no cytokine responses to LT [3]. These striking effects could be the result of direct targeting of cardiomyocytes or heart endothelium by toxin, or once again secondary to LT effects on other cells controlling peripheral mediators of vasculature homeostasis. The altered architecture and vacuolation of endothelial cells, however, is suggestive of direct LT effects on these cells in the heart. It is important to note that as these LT-induced changes were only observed by electron microscopy, it is possible that LT also affects endothelial cells in many other target organs in a similar manner and even at earlier times. Furthermore, the LT effects reported here are in the context of a bolus toxin injection model, and it is more than likely that *B. anthracis* infections would target numerous organs earlier during the course of disease, before LT levels affecting the heart or endothelium are reached. Nonetheless, the heart damage induced by LT, as measured by rapid and extreme release of cardiac biomarkers, is the earliest currently known *in vivo* effect of this toxin in mice.

An interesting finding in this work is the clear beneficial effect of nNOS in LT-mediated pathogenesis, while iNOS and eNOS appear to play no role. NO is a key regulator of vascular function, affecting blood pressure and flow regulation, platelet aggregation, and leukocyte adhesion, among others. Although its designation implies preferential expression in neural tissues, nNOS is actually expressed in skeletal muscle, smooth muscle, and the heart, as well as in neurons [54]. However, nNOS-derived NO is believed to primarily manifest its most potent protective effects in the heart (for reviews see [19]–[21],[55],[56]) and it is possible that the protective effects of this enzyme after LT treatment are also based on its function in the heart. All effects of the NO in the heart are localized paracrine effects dependent on the location of eNOS and nNOS (the two constitutive enzymes in the heart). This is because of the high content of myoglobin in the heart, which acts as a quencher of NO and thus does not allow this normally highly diffusible molecule to function at distances in the heart. In the heart eNOS is associated with the caveolae [29],[57] and nNOS is normally in the sarcoplasmic reticulum (SR) in close association with ryanodine receptors [29],[58] and XOR [37],[38]. nNOS-derived NO mediates cardiomyocyte contraction through its effects on the SR Ca^+2^ cycle that is central to excitation-contraction coupling and modulation of ryanodine receptor function [21], [30]–[32],[59]. NO produced by nNOS is also cardioprotective by functioning as a local antioxidant which inactivates the superoxide produced constitutively by the co-localized XOR [36]–[38]. As a result, nNOS−/− mice have 60% higher superoxide production than WT mice, and this increased ROS depresses myocardial contractility [38]. We found, however, that inhibition of XOR had a detrimental effect on survival of LT-treated WT mice. Interestingly, XOR inhibition with allopurinol was also unable to reverse the increased susceptibility of nNOS−/− mice to this toxin (data not shown) indicating that nNOS was unlikely to be manifesting its protective functions through reversing XOR-produced effects.

Multiple explanations can be offered for the protective role of nNOS against LT. Perhaps the simpler view is that nNOS deficiency results in an imbalance between oxidative stress and NO signaling such that the ROS status of the heart leaves it more susceptible to LT-induced damage. Alternatively, nNOS-derived NO could be acting to maintain normal cardiomyocyte function, which serves to counteract the toxin's deleterious effects on cardiomyocytes or the peripheral vasculature. In the absence of nNOS, animals cannot respond as well to LT-mediated cardiac dysfunction, much as has been noted for other cardiac injury models. In fact, during cardiac stress or heart failure nNOS has been shown to translocate to the sarcolemma [60]–[62] and this relocalization of nNOS results in a decreased ability to control contractility via ryanodine receptor [20],[21],[55]. nNOS and its activity are upregulated and protective in heart failure. As a result a higher percentage of nNOS−/− mice die after induction of myocardial infarction [60]–[63].

Despite the appeal of the hypothesis that nNOS protects against LT by providing improved contractile function in response to direct or indirect cardiomyocyte damage, it must be noted that virtually all the reports on the effects of nNOS deficiency on heart myocytes are contradictory. Basal increase, decrease and lack of effect on contractility function have been reported for nNOS−/− mouse myocytes [29]–[32]. nNOS deficiency effects on ryanodine receptors in vitro are also contradictory [30],[64],[65]. Even what were deemed as the definitive experiments to resolve these conflicts – namely the tissue-specific over-expression of nNOS in myocardial tissue, resulted in reduced contractility and reduced ejection fraction (EF) in one study [66] and increased contractility and protective function in another [67]. A newer set of MRI studies on heart function indicated that nNOS does not play a role in basal contractility, but its absence can have severe effects for β-adrenergic responses in the heart [68]. It is important to note that despite the shown LT effects on heart function in LT-sensitive rats, the potent 7-NI nNOS inhibitor was unable to sensitize LT-resistant Lewis rats at multiple doses (data not shown), indicating a genetic-based resistance in rats that differed from the sensitization seen in mice.

nNOS-derived NO may also provide a protective function against LT through its control of mitochondrial respiration. Mitochondrial degeneration was seen in response to LT in mouse hearts (Figure 4, Figure 6). NO is an inhibitor of cytochrome oxidase and can act as an antioxidant, protecting against LT-mediated mitochondrial ROS production [69]. In fact, nNOS−/− mice have been shown to have altered respiratory responses during hypoxia [70] and we know LT induces hypoxia secondary to vascular collapse [3]. nNOS deletion from the nervous systems or blood vessels may also result in increased sensitivity to LT. nNOS has been shown to provide vasculoprotective function at the vessel level [71],[72] and to control microvascular tone [73]. nNOS protective function in endotoxin sepsis, for example, was postulated to occur through altered cytokine regulation of the vasculature [14]. Clearly nNOS may provide protection against LT at multiple tissue sites or due to effects in vasculature in all organs.

The molecular mechanism(s) by which LT induces the rapid pathological changes described in this report and the associated rapid release of cardiac biomarkers (in < 6 h) is unknown. These are the earliest events yet observed in C57BL/6J mice in response to LT treatment in the bolus challenge model. The direct inactivation of key MAPK pathways by the lethal factor protease may initiate these events. Activation of the MEK1/2 pathway is considered a protective response in the heart and has been clearly linked to cardiac hypertrophy in many independent studies, including those where overexpression of activated MEK1 in transgenic mice results in dramatic increases in cardiac contractile performance (for review, see [74]). Furthermore, it has been reported that the MEK1 pathway is involved in upregulation of nNOS transcription under some circumstances [75]. With the known strong links between MEK1/2 activation and cardioprotection, it would be interesting to know whether cleavage and inactivation of MEK1/2 by LT in the heart leads directly to a loss of nNOS function. Interestingly, there are several examples of acute myocarditis and sudden death as a result of rapid proteolytic cleavage events in the heart caused by infectious agents. Coxsackie virus destroys cardiomyocytes when its protease 2A cleaves dystrophin in these cells [76],[77] and cardiac NO actually protects against this cleavage [78]. A recent study reported LT-mediated breakdown of nNOS in an LT-sensitive macrophage cell line after toxin treatment [79]. However these dying cells also have the breakdown of numerous other proteins and the *in vitro* cleavage of nNOS by LT in this study required very high ratios of enzyme to substrate. In this study nNOS activity was also unaffected by LT, leaving the interpretation of the reported findings uncertain.

Protection against anthrax disease is absolute with neutralizing antibody against the PA component of anthrax lethal toxin alone. Thus anthrax toxin is still considered the major relevant virulence factor for this disease and understanding the mechanisms by which LT causes mortality and vascular insufficiency can aid in unraveling the mystery of how this disease kills. We believe the LT-mediated cardiopathology described here is a step toward a better understanding of this toxin's effects in vivo. Our future studies will focus on deciphering the role the cleavage and inactivation of MEK proteins may or may not play in LT-induced cardiomyopathy, the molecular mechanism of LT-induced early cardiac damage, the means by which nNOS-derived NO is protective against LT effects and the tissue sites at which nNOS manifests these effects.

## Materials and Methods

### Materials

LF and PA were purified from *B. anthracis* as previously described [80]. LF used in this study, like that in all our prior work, is a recombinant variant having the N-terminal sequence HMAGG [4]. Toxin was prepared in sterile PBS for animal injections and in serum-free Dulbecco's modified Eagle medium (DMEM) (Invitrogen, Carslbad, CA) for cytotoxicity studies. LT doses and concentrations correspond to equivalent amounts of each toxin component (i.e., 100 µg LT is 100 µg PA plus 100 µg LF). 7-nitroindazole, sodium salt (7-NI, Calbiochem, San Diego, CA), allopurinol (TCI America, Portland, OR), carboxy-PTIO (2-phenyl-4,4,5,5,-tetramethylimidazoline-1-oxyl-3-oxide, Cayman Chemical Company, Ann Arbor, MI) and sodium nitrite (Mallinckrodt Baker, Phillipsburg, NJ) were used at various doses as described in each experiment.

### Animal studies

C57BL/6J, B6.129SF2/J, and B6.129S4-*Nos1^tm1Plh^*/J (nNOS−/−) mice (8–12 weeks old, female, 20–25 g) were purchased from Jackson Laboratories (Bar Harbor, Maine). Age-matched mice were used for all experiments. For survival studies, animals were injected intraperitoneally (IP) or intravenously (IV) with drugs or toxin according to described schedules and observed for malaise symptoms over time. For cytokine or cardiac biomarker measurements, mice were bled at various times after LT injection by cardiac puncture under anesthesia using microvette serum separator tubes (Sarstedt Inc., Newton, NC). Mouse tissues (spleen, liver, brain, kidney, heart, lung, femoral bone) were harvested for histopathological analyses at various times after LT administration (100 µg IP) and fixed in 10% neutral buffered formalin, embedded in paraffin and stained with hematoxylin and eosin (H&E). Selected heart sections were stained by Masson's trichrome or immunostained with anti-myeloperoxidase (DakoCytomation, Glostrup, Denmark and Abcam, Cambridge, MA), anti-nNOS (Zymed Laboratories, San Francisco, CA) and anti-cytochrome c oxidase I (Santa Cruz Biotech, Santa Cruz, CA). Terminal deoxynucleotidyl transferase (TdT)-mediated dUTP-digoxigenin nick end-labeling (TUNEL) was performed on some heart sections. Staining procedures were performed by Histoserv Inc. (Gaithersburg, MD). For electron microscopy, heart sections were fixed in 4% paraformaldehyde/2.5% glutaraldehyde (Electron Microscopy Sciences, Hatfield, PA) made in PBS containing 0.05 M sucrose. Tissues were washed twice in 0.1 M sodium phosphate buffer, pH 7.2, and post fixed in 1% osmium tetroxide in the same buffer. Following one phosphate buffer wash and two washes with deionized water, samples were stained with 1% uranyl acetate, dehydrated in ethanol, and embedded in Spurr's low viscosity resin (Ted Pella, Inc., Redding, CA). Sections were cut with a diamond knife and examined at 80 kV, with a model H7500 transmission electron microscope (Hitachi High Technologies, America, Pleasanton, CA). Digital images were collected with an XR-100 camera (Advanced Microscopy Techniques, Inc., Danvers, MA). For echocardiography studies, mice were kept under isoflurane anesthesia on a heating pad with body temperature monitoring (by anal probe) throughout the procedure. Baseline left ventricular short and long axis views were obtained with a 30 MHZ RMV 707B mechanical sector transducer (Visualsonics Inc., Toronto, Canada), with a minimum of 3 images recorded/animal. All measurements were performed in blinded fashion.

### Cytotoxicity assays

Bone marrow-derived macrophages (BMDMs) from mice were cultured in differentiation medium consisting of two parts complete DMEM (10% fetal bovine serum, 10 mM Hepes, and 50 µg/ml gentamicin) and one part L929 cell culture supernatant (also grown in complete DMEM) for 7 days prior to plating in 96-well plates for cytotoxicity testing.

### Cytokine and cardiac biomarker measurements

ELISA kits for murine IL-1β, MIP1 and IL-6 (R&D systems, Minneapolis, MN) were utilized for assessment of cytokine levels. Murine cTroponin-I (cTnI), heart-type fatty acid binding protein (H-FABP), and myoglobin ELISA kits (Life Diagnostics, West Chester, PA) were used to measure these cardiac biomarkers in serum samples. All kits were used according to manufacturer's protocols.

## Supporting Information

Figure S1 Echocardiography of LT-treated nNOS−/− and WT mouse heart. Mice were treated with LT (100 µg IP) and echocardiography performed at 24–28 h post toxin administration. Each symbol represents measurements for one mouse and the mean for each group is also shown. Panels show the following parameters as measured for LT-treated and untreated knockout and WT mice: Ds and Dd are the left ventricle end systolic and diastolic measurements, respectively; Vs and Vd are the left ventricle-end systolic and diastolic volumes; SV is stroke volume; EF is ejection fraction; FS is the fractional shortening; CO is the cardiac output. Left ventricle Vs and Ds differed significantly between the nNOS−/− and WT mice (P = 0.0202 and P = 0.0176, respectively). Ejection fraction (EF) and fractional shortening (FS) parameters were also different between these mice (P = 0.0325 and 0.0368, respectively). LT-treated groups were not statistically different from their untreated counterparts for any parameter but a decrease in EF values following toxin treatment was indicative of contractile dysfunction.
(0.02 MB PDF)

## References

[1] Leppla SH, Alouf JE, Popoff MR (2006). *Bacillus anthracis* toxins.. The Comprehensive Sourcebook of Bacterial Protein Toxins.

[2] Moayeri M, Martinez NW, Wiggins J, Young HA, Leppla SH (2004). Mouse susceptibility to anthrax lethal toxin is influenced by genetic factors in addition to those controlling macrophage sensitivity.. Infect Immun.

[3] Moayeri M, Haines D, Young HA, Leppla SH (2003). *Bacillus anthracis* lethal toxin induces TNF-alpha-independent hypoxia-mediated toxicity in mice.. J Clin Invest.

[4] Gupta PK, Moayeri M, Crown D, Fattah RJ, Leppla SH (2008). Role of N-terminal amino acids in the potency of anthrax lethal factor.. PLoS ONE.

[5] Ezzell JW, Ivins BE, Leppla SH (1984). Immunoelectrophoretic analysis, toxicity, and kinetics of in vitro production of the protective antigen and lethal factor components of *Bacillus anthracis* toxin.. Infect Immun.

[6] Duesbery NS, Webb CP, Leppla SH, Gordon VM, Klimpel KR (1998). Proteolytic inactivation of MAP-kinase-kinase by anthrax lethal factor.. Science.

[7] Vitale G, Bernardi L, Napolitani G, Mock M, Montecucco C (2000). Susceptibility of mitogen-activated protein kinase kinase family members to proteolysis by anthrax lethal factor.. Biochem J.

[8] Kalns J, Scruggs J, Millenbaugh N, Vivekananda J, Shealy D (2002). TNF receptor 1, IL-1 receptor, and iNOS genetic knockout mice are not protected from anthrax infection.. Biochem Biophys Res Commun.

[9] Cui X, Moayeri M, Li Y, Li X, Haley M (2004). Lethality during continuous anthrax lethal toxin infusion is associated with circulatory shock but not inflammatory cytokine or nitric oxide release in rats.. Am J Physiol Regul Integr Comp Physiol.

[10] Raines KW, Kang TJ, Hibbs S, Cao GL, Weaver J (2006). Importance of nitric oxide synthase in the control of infection by *Bacillus anthracis*.. Infect Immun.

[11] Pellizzari R, Guidi-Rontani C, Vitale G, Mock M, Montecucco C (2000). Lethal factor of *Bacillus anthracis* cleaves the N-terminus of MAPKKs: analysis of the intracellular consequences in macrophages.. Int J Med Microbiol.

[12] Kuo SR, Willingham MC, Bour SH, Andreas EA, Park SK (2008). Anthrax toxin-induced shock in rats is associated with pulmonary edema and hemorrhage.. Microb Pathog.

[13] Gozes Y, Moayeri M, Wiggins JF, Leppla SH (2006). Anthrax lethal toxin induces ketotifen-sensitive intradermal vascular leakage in certain inbred mice.. Infect Immun.

[14] Cui X, Besch V, Khaibullina A, Hergen A, Quezado M (2007). Neuronal nitric oxide synthase deficiency decreases survival in bacterial peritonitis and sepsis.. Intensive Care Med.

[15] Razavi HM, Hamilton JA, Feng Q (2005). Modulation of apoptosis by nitric oxide: implications in myocardial ischemia and heart failure.. Pharmacol Ther.

[16] Andreka P, Tran T, Webster KA, Bishopric NH (2004). Nitric oxide and promotion of cardiac myocyte apoptosis.. Mol Cell Biochem.

[17] Ward ME, Toporsian M, Scott JA, Teoh H, Govindaraju V (2005). Hypoxia induces a functionally significant and translationally efficient neuronal NO synthase mRNA variant.. J Clin Invest.

[18] Comini L, Boraso A, Bachetti T, Bernocchi P, Pasini E (2005). Effects of endotoxic shock on neuronal NOS and calcium transients in rat cardiac myocytes.. Pharmacol Res.

[19] Casadei B (2006). The emerging role of neuronal nitric oxide synthase in the regulation of myocardial function.. Exp Physiol.

[20] Loyer X, Samuel JL, Heymes C (2005). Cardiac myocyte neuronal nitric oxide synthase. New therapeutic target in heart failure?. Arch Mal Coeur Vaiss.

[21] Sears CE, Ashley EA, Casadei B (2004). Nitric oxide control of cardiac function: is neuronal nitric oxide synthase a key component?. Philos Trans R Soc Lond B Biol Sci.

[22] Plebani M, Zaninotto M (1998). Diagnostic strategies in myocardial infarction using myoglobin measurement.. Eur Heart J.

[23] O'Brien PJ (2008). Cardiac troponin is the most effective translational safety biomarker for myocardial injury in cardiotoxicity.. Toxicology.

[24] O'Brien PJ (2006). Blood cardiac troponin in toxic myocardial injury: archetype of a translational safety biomarker.. Expert Rev Mol Diagn.

[25] Babuin L, Jaffe AS (2005). Troponin: the biomarker of choice for the detection of cardiac injury.. CMAJ.

[26] O'Brien PJ, Smith DE, Knechtel TJ, Marchak MA, Pruimboom-Brees I (2006). Cardiac troponin I is a sensitive, specific biomarker of cardiac injury in laboratory animals.. Lab Anim.

[27] Colli A, Josa M, Pomar JL, Mestres CA, Gherli T (2007). Heart fatty acid binding protein in the diagnosis of myocardial infarction: where do we stand today?. Cardiology.

[28] Alhadi HA, Fox KA (2004). Do we need additional markers of myocyte necrosis: the potential value of heart fatty-acid-binding protein.. QJM.

[29] Barouch LA, Harrison RW, Skaf MW, Rosas GO, Cappola TP (2002). Nitric oxide regulates the heart by spatial confinement of nitric oxide synthase isoforms.. Nature.

[30] Sears CE, Bryant SM, Ashley EA, Lygate CA, Rakovic S (2003). Cardiac neuronal nitric oxide synthase isoform regulates myocardial contraction and calcium handling.. Circ Res.

[31] Ashley EA, Sears CE, Bryant SM, Watkins HC, Casadei B (2002). Cardiac nitric oxide synthase 1 regulates basal and beta-adrenergic contractility in murine ventricular myocytes.. Circulation.

[32] Khan SA, Skaf MW, Harrison RW, Lee K, Minhas KM (2003). Nitric oxide regulation of myocardial contractility and calcium cycling: independent impact of neuronal and endothelial nitric oxide synthases.. Circ Res.

[33] Sweeney D, Cui X, Solomon S, Subramanian M, Li Y (2008). Anthrax edema and lethal toxins produce very different patterns of cardiovascular dysfunction in canines.. Am J Respir Crit Care Med.

[34] Berry CE, Hare JM (2004). Xanthine oxidoreductase and cardiovascular disease: molecular mechanisms and pathophysiological implications.. J Physiol.

[35] Harrison R (2004). Physiological roles of xanthine oxidoreductase.. Drug Metab Rev.

[36] Zhang GX, Kimura S, Murao K, Shimizu J, Matsuyoshi H (2008). Role of neuronal NO synthase in regulating vascular superoxide levels and mitogen-activated protein kinase phosphorylation.. Cardiovasc Res.

[37] Kinugawa S, Huang H, Wang Z, Kaminski PM, Wolin MS (2005). A defect of neuronal nitric oxide synthase increases xanthine oxidase-derived superoxide anion and attenuates the control of myocardial oxygen consumption by nitric oxide derived from endothelial nitric oxide synthase.. Circ Res.

[38] Khan SA, Lee K, Minhas KM, Gonzalez DR, Raju SV (2004). Neuronal nitric oxide synthase negatively regulates xanthine oxidoreductase inhibition of cardiac excitation-contraction coupling.. Proc Natl Acad Sci U S A.

[39] Kittleson MM, Hare JM (2005). Xanthine oxidase inhibitors: an emerging class of drugs for heart failure.. Eur Heart J.

[40] Boueiz A, Damarla M, Hassoun PM (2008). Xanthine oxidoreductase in respiratory and cardiovascular disorders.. Am J Physiol Lung Cell Mol Physiol.

[41] Sinha SS, Shiva S, Gladwin MT (2008). Myocardial protection by nitrite: evidence that this reperfusion therapeutic will not be lost in translation.. Trends Cardiovasc Med.

[42] Dezfulian C, Raat N, Shiva S, Gladwin MT (2007). Role of the anion nitrite in ischemia-reperfusion cytoprotection and therapeutics.. Cardiovasc Res.

[43] Shiva S, Sack MN, Greer JJ, Duranski M, Ringwood LA (2007). Nitrite augments tolerance to ischemia/reperfusion injury via the modulation of mitochondrial electron transfer.. J Exp Med.

[44] Kevil CG, Patel RP (2008). Preserving vessel function during ischemic disease: new possibilities of inorganic nitrite therapy.. Expert Rev Cardiovasc Ther.

[45] Carson-Walter EB, Watkins DN, Nanda A, Vogelstein B, Kinzler KW (2001). Cell surface tumor endothelial markers are conserved in mice and humans.. Cancer Res.

[46] Bell SE, Mavila A, Salazar R, Bayless KJ, Kanagala S (2001). Differential gene expression during capillary morphogenesis in 3D collagen matrices: regulated expression of genes involved in basement membrane matrix assembly, cell cycle progression, cellular differentiation and G-protein signaling.. J Cell Sci.

[47] Liu S, Wang H, Currie BM, Molinolo A, Leung HJ (2008). Matrix metalloproteinase-activated anthrax lethal toxin demonstrates high potency in targeting tumor vasculature.. J Biol Chem.

[48] Kirby JE (2004). Anthrax lethal toxin induces human endothelial cell apoptosis.. Infect Immun.

[49] Pandey J, Warburton D (2004). Knock-on effect of anthrax lethal toxin on macrophages potentiates cytotoxicity to endothelial cells.. Microbes Infect.

[50] Warfel JM, Steele AD, D'Agnillo F (2005). Anthrax lethal toxin induces endothelial barrier dysfunction.. Am J Pathol.

[51] Watson LE, Kuo SR, Katki K, Dang T, Park SK (2007). Anthrax toxins induce shock in rats by depressed cardiac ventricular function.. PLoS ONE.

[52] Sherer K, Li Y, Cui X, Li X, Subramanian M (2007). Fluid support worsens outcome and negates the benefit of protective antigen-directed monoclonal antibody in a lethal toxin-infused rat *Bacillus anthracis* shock model.. Crit Care Med.

[53] Cui X, Li Y, Moayeri M, Choi GH, Subramanian GM (2005). Late treatment with a protective antigen-directed monoclonal antibody improves hemodynamic function and survival in a lethal toxin-infused rat model of anthrax sepsis.. J Infect Dis.

[54] Mungrue IN, Bredt DS (2004). nNOS at a glance: implications for brain and brawn.. J Cell Sci.

[55] Lim G, Venetucci L, Eisner DA, Casadei B (2008). Does nitric oxide modulate cardiac ryanodine receptor function? Implications for excitation-contraction coupling.. Cardiovasc Res.

[56] Loyer X, Heymes C, Samuel JL (2008). Constitutive nitric oxide synthases in the heart from hypertrophy to failure.. Clin Exp Pharmacol Physiol.

[57] Feron O, Belhassen L, Kobzik L, Smith TW, Kelly RA (1996). Endothelial nitric oxide synthase targeting to caveolae. Specific interactions with caveolin isoforms in cardiac myocytes and endothelial cells.. J Biol Chem.

[58] Xu KY, Huso DL, Dawson TM, Bredt DS, Becker LC (1999). Nitric oxide synthase in cardiac sarcoplasmic reticulum.. Proc Natl Acad Sci U S A.

[59] Seddon M, Shah AM, Casadei B (2007). Cardiomyocytes as effectors of nitric oxide signalling.. Cardiovasc Res.

[60] Damy T, Ratajczak P, Shah AM, Camors E, Marty I (2004). Increased neuronal nitric oxide synthase-derived NO production in the failing human heart.. Lancet.

[61] Damy T, Ratajczak P, Robidel E, Bendall JK, Oliviero P (2003). Up-regulation of cardiac nitric oxide synthase 1-derived nitric oxide after myocardial infarction in senescent rats.. FASEB J.

[62] Bendall JK, Damy T, Ratajczak P, Loyer X, Monceau V (2004). Role of myocardial neuronal nitric oxide synthase-derived nitric oxide in beta-adrenergic hyporesponsiveness after myocardial infarction-induced heart failure in rat.. Circulation.

[63] Dawson D, Lygate CA, Zhang MH, Hulbert K, Neubauer S (2005). nNOS gene deletion exacerbates pathological left ventricular remodeling and functional deterioration after myocardial infarction.. Circulation.

[64] Stoyanovsky D, Murphy T, Anno PR, Kim YM, Salama G (1997). Nitric oxide activates skeletal and cardiac ryanodine receptors.. Cell Calcium.

[65] Zahradnikova A, Minarovic I, Venema RC, Meszaros LG (1997). Inactivation of the cardiac ryanodine receptor calcium release channel by nitric oxide.. Cell Calcium.

[66] Burkard N, Rokita AG, Kaufmann SG, Hallhuber M, Wu R (2007). Conditional neuronal nitric oxide synthase overexpression impairs myocardial contractility.. Circ Res.

[67] Loyer X, Gomez AM, Milliez P, Fernandez-Velasco M, Vangheluwe P (2008). Cardiomyocyte overexpression of neuronal nitric oxide synthase delays transition toward heart failure in response to pressure overload by preserving calcium cycling.. Circulation.

[68] Vandsburger MH, French BA, Helm PA, Roy RJ, Kramer CM (2007). Multi-parameter in vivo cardiac magnetic resonance imaging demonstrates normal perfusion reserve despite severely attenuated beta-adrenergic functional response in neuronal nitric oxide synthase knockout mice.. Eur Heart J.

[69] Brown GC, Borutaite V (2007). Nitric oxide and mitochondrial respiration in the heart.. Cardiovasc Res.

[70] Kline DD, Yang T, Huang PL, Prabhakar NR (1998). Altered respiratory responses to hypoxia in mutant mice deficient in neuronal nitric oxide synthase.. J Physiol.

[71] Tsutsui M (2004). Neuronal nitric oxide synthase as a novel anti-atherogenic factor.. J Atheroscler Thromb.

[72] Morishita T, Tsutsui M, Shimokawa H, Horiuchi M, Tanimoto A (2002). Vasculoprotective roles of neuronal nitric oxide synthase.. FASEB J.

[73] Seddon MD, Chowienczyk PJ, Brett SE, Casadei B, Shah AM (2008). Neuronal nitric oxide synthase regulates basal microvascular tone in humans in vivo.. Circulation.

[74] Bueno OF, Molkentin JD (2002). Involvement of extracellular signal-regulated kinases 1/2 in cardiac hypertrophy and cell death.. Circ Res.

[75] Nakata S, Tsutsui M, Shimokawa H, Tamura M, Tasaki H (2005). Vascular neuronal NO synthase is selectively upregulated by platelet-derived growth factor: involvement of the MEK/ERK pathway.. Arterioscler Thromb Vasc Biol.

[76] Badorff C, Lee GH, Lamphear BJ, Martone ME, Campbell KP (1999). Enteroviral protease 2A cleaves dystrophin: evidence of cytoskeletal disruption in an acquired cardiomyopathy.. Nat Med.

[77] Knowlton KU (2008). CVB infection and mechanisms of viral cardiomyopathy.. Curr Top Microbiol Immunol.

[78] Badorff C, Fichtlscherer B, Rhoads RE, Zeiher AM, Muelsch A (2000). Nitric oxide inhibits dystrophin proteolysis by coxsackieviral protease 2A through S-nitrosylation: A protective mechanism against enteroviral cardiomyopathy.. Circulation.

[79] Kim J, Park H, Chang M, Han SH, Chung H (2008). The effects of anthrax lethal factor on the macrophage proteome: Potential activity on nitric oxide synthases.. Arch Biochem Biophys.

[80] Varughese M, Chi A, Teixeira AV, Nicholls PJ, Keith JM (1998). Internalization of a *Bacillus anthracis* protective antigen-c-Myc fusion protein mediated by cell surface anti-c-Myc antibodies.. Mol Med.

