# Supplementary figures and images for: The Heart Is an Early Target of Anthrax Lethal Toxin in Mice: A Protective Role for Neuronal Nitric Oxide Synthase (nNOS)
Source: PLoS Pathog. 2009 May 29;5(5):e1000456. doi: 10.1371/journal.ppat.1000456 (PMC2680977; doi:10.1371/journal.ppat.1000456)

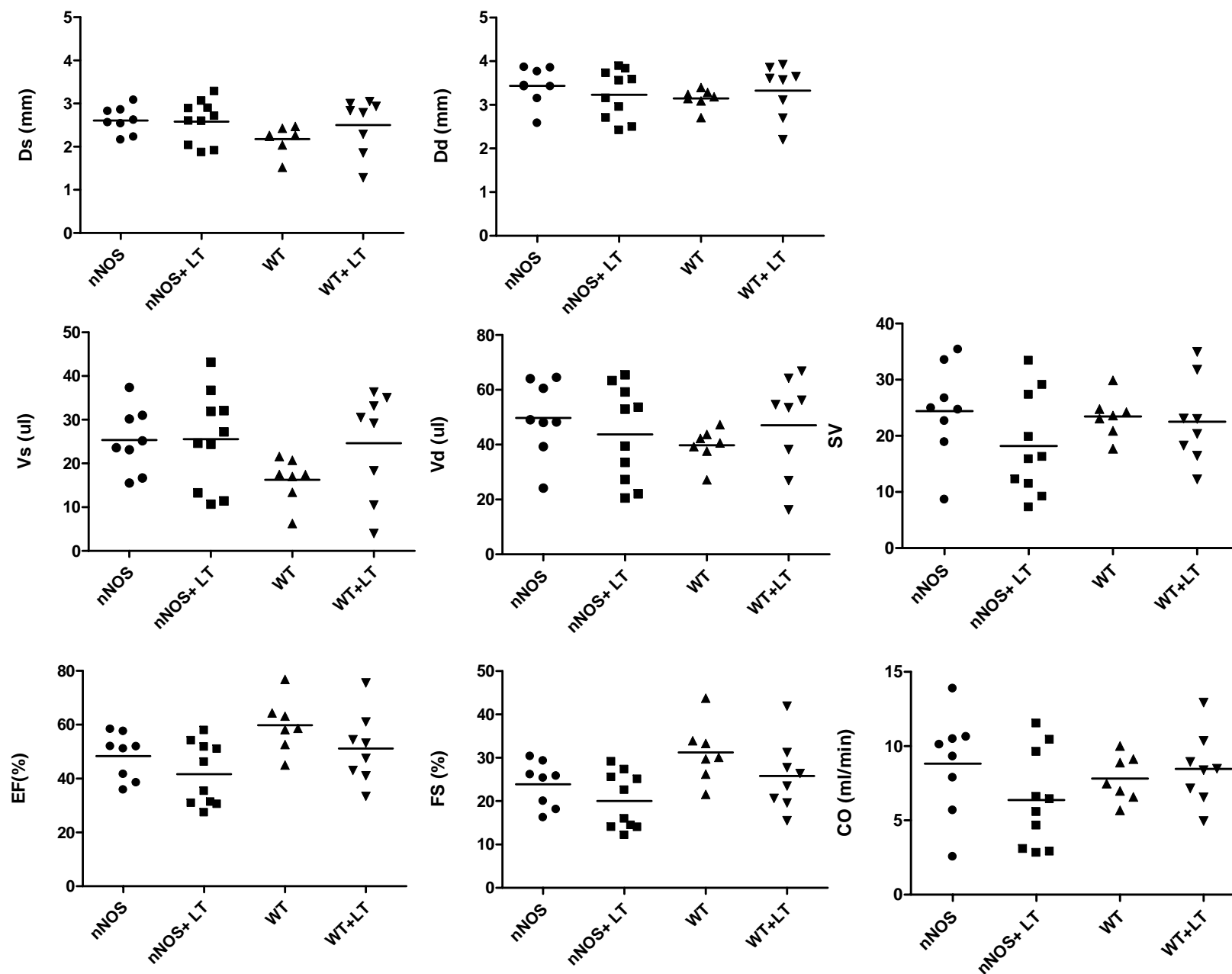

Supplemental Fig. 1

Supplement: Figure S1 — Echocardiography of LT-treated nNOS−/− and WT mouse heart. Mice were treated with LT (100 µg IP) and echocardiography performed at 24–28 h post toxin administration. Each symbol represents measurements for one mouse and the mean for each group is also shown. Panels show the following parameters as measured for LT-treated and untreated knockout and WT mice: Ds and Dd are the left ventricle end systolic and diastolic measurements, respectively; Vs and Vd are the left ventricle-end systolic and diastolic volumes; SV is stroke volume; EF is ejection fraction; FS is the fractional shortening; CO is the cardiac output. Left ventricle Vs and Ds differed significantly between the nNOS−/− and WT mice (P = 0.0202 and P = 0.0176, respectively). Ejection fraction (EF) and fractional shortening (FS) parameters were also different between these mice (P = 0.0325 and 0.0368, respectively). LT-treated groups were not statistically different from their untreated counterparts for any parameter but a decrease in EF values following toxin treatment was indicative of contractile dysfunction. (0.02 MB PDF) [file ppat.1000456.s001.pdf]
